# Highly-conserved regulatory activity of the ANR family in the virulence of diarrheagenic bacteria through interaction with master and global regulators

**DOI:** 10.1038/s41598-023-33997-0

**Published:** 2023-04-29

**Authors:** Diana Rodriguez-Valverde, Jorge A. Giron, Yang Hu, James P. Nataro, Fernando Ruiz-Perez, Araceli E. Santiago

**Affiliations:** 1 Child Health Research Center, Department of Pediatrics, University of Virginia School of Medicine, 409 Lane Road, MR-4 Building, P.O. Box 801326, Charlottesville, VA 22908 USA; 2 Centro de Detección Biomolecular, Benemérita Universidad Autónoma de Puebla, Puebla, Mexico; 3 Translational Genomics Research Institute, 445 N. 5th St, Phoenix, AZ 85004 USA; 4 CD Genomics, Shirley, NY USA

**Keywords:** Immunopathogenesis, Bacteria, Infectious-disease diagnostics, Microbiology, Diseases, Pathogenesis

## Abstract

ANR (AraC negative regulators) are a novel class of small regulatory proteins commonly found in enteric pathogens. Aar (AggR-activated regulator), the best-characterized member of the ANR family, regulates the master transcriptional regulator of virulence AggR and the global regulator HNS in enteroaggregative *Escherichia coli* (EAEC) by protein–protein interactions. On the other hand, Rnr (RegA-negative regulator) is an ANR homolog identified in attaching and effacing (AE) pathogens, including *Citrobacter rodentium* and enteropathogenic *Escherichia coli* (EPEC), sharing only 25% identity with Aar. We previously found that *C. rodentium* lacking Rnr exhibits prolonged shedding and increased gut colonization in mice compared to the parental strain. To gain mechanistic insights into this phenomenon, we characterized the regulatory role of Rnr in the virulence of prototype EPEC strain E2348/69 by genetic, biochemical, and human organoid-based approaches. Accordingly, RNA-seq analysis revealed more than 500 genes differentially regulated by Rnr, including the type-3 secretion system (T3SS). The abundance of EspA and EspB in whole cells and bacterial supernatants confirmed the negative regulatory activity of Rnr on T3SS effectors. We found that besides HNS and Ler, twenty-six other transcriptional regulators were also under Rnr control. Most importantly, the deletion of *aar* in EAEC or *rnr* in EPEC increases the adherence of these pathogens to human intestinal organoids. In contrast, the overexpression of ANR drastically reduces bacterial adherence and the formation of AE lesions in the intestine. Our study suggests a conserved regulatory mechanism and a central role of ANR in modulating intestinal colonization by these enteropathogens despite the fact that EAEC and EPEC evolved with utterly different virulence programs.

## Introduction

The mortality and morbidity associated with enteric infections remain a significant public health problem worldwide. EPEC and EAEC are etiologic agents of diarrhea in developed and developing countries^1–10^. The hallmark of EPEC infection is the formation of attaching and effacing (A/E) lesions in the intestinal epithelium, characterized by a localized accumulation of F-actin and effacement of the brush border microvilli upon intimate bacterial attachment to the apical plasma membrane (pedestal formation)^2,11^, and which depends on the T3SS encoded in the LEE PAI^12,13^. The T3SS resembles a needle-like structure formed by several proteins, in which filaments of the needle are made of EspA, and the tip of the needle contains EspB and EspD forming a pore inserted into the host cell membrane^14–17^. Pedestal formation is initiated by the secretion of the translocated-intimin receptor (Tir) and its incorporation into the host cell plasma membrane. Upon proximity of the bacterium to the host cell, Tir interacts with the bacterial outer membrane protein intimin and initiates a signaling cascade involving tyrosine kinases and cytoskeletal changes^18–20^. Typical EPEC also shows a “localized adherence" phenotype (LA), which is dependent on the type IV bundle-forming pilus (BFP)^21^. The expression of the LEE is subjected to various levels of regulation, including feedback inhibition, transcriptional activation, and transcriptional repression.

On the other hand, EAEC pathogenesis comprises colonization of small and large intestinal mucosal surfaces, mainly mediated by the aggregative adherence fimbriae (AAF) and the elaboration of enterotoxins and cytotoxins that damage host cells, inducing inflammation and diarrhea^1,3,6,22^. AggR, an AraC/XylS family activator, is the master regulator of virulence in EAEC. It controls the expression of at least 44 genes, including the AAF required for bacterial adherence^23,24^, the dispersin lipoprotein Aap^25^, the dispersin secretion system AAT^26^, the non-canonical *N*-acyltransferase AatD^27^ and a chromosomally encoded type VI secretion system called AAI^23,28^.

We recently identified the ANR (AraC Negative Regulators), a large family of small (< 10 kDa) regulatory proteins produced by diverse clinically significant enteric pathogens, including *Vibrio* spp., *Salmonella* spp., *Shigella* spp., *Yersinia* spp., *Citrobacter* spp., and pathogenic *E. coli* such as enterotoxigenic *E. coli* (ETEC), EAEC, and EPEC*.* Aar (AggR-activated regulator), the best-characterized member of the ANR family of EAEC, modulates the expression of at least 200 genes associated with fitness and virulence by interfering with the function of AraC/XylS positive transcriptional regulators and HNS global repressors through protein–protein interactions, consequently preventing their binding to DNA^29–31^.

We also identified an Aar homolog in *C. rodentium* (CR) termed Rnr (RegA negative regulator), which is highly conserved in other AE pathogens. The CR*rnr* mutant exhibits high levels of expression of RegA and RegA-regulated fimbrial Kfc and increased gut colonization compared to the parental strain^29^. To gain mechanistic insights into this phenotype, in this study, we sought to determine the Rnr regulatory mechanisms governing the expression of virulence factors in the epidemiologically relevant EPEC pathogen and its relevance in the context of intestinal colonization, as a prerequisite to the development of strategies to prevent and treat EPEC infections, which can also apply to other pathogens**.**

## Results

### ANR is relatively conserved among diarrheagenic pathogens

**Figure 1:**
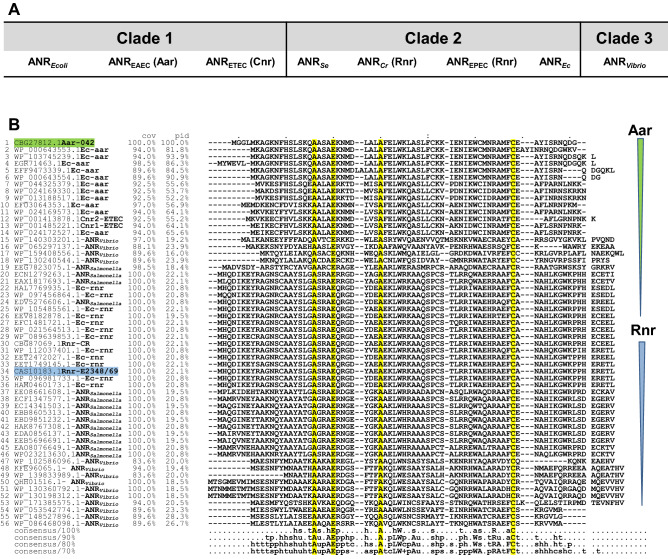
Phylogenetic analysis of the ANR family in diarrheagenic bacteria. The nomenclature used for ANR members in each Clade is shown in (**A**). Phylogenetic analysis of the amino acid sequence of previous and newly identified ANR members from pathogenic *E. coli*,* Salmonella*, and *Vibrio* species was carried out using the Clustalw algorithm. The percentage of protein identity/similarities among ANRs is shown in (**B**). Highly conserved amino acids in the family are highlighted.

Over the last 5 years, massive sequencing of new bacterial genomes has identified hundreds of new ANR members in multiple pathogens. ANR is widely distributed in at least 26 Gram-negative bacterial species^29,31^. Phylogenetic analysis of the amino acid sequence of ANR members from clinically relevant diarrheagenic bacteria, such as pathogenic *E. coli*, *Salmonella enterica*, and *Vibrio *sp., revealed divergence in ANR cognates that fall in at least three clades (termed 1 to 3) (Fig. 1A). The archetype ANR, Aar (from EAEC) and Cnr (from ETEC) fall in Clade-1. ANR homologs from AE pathogens such as EPEC, *C. rodentium* (Rnr), and *Salmonella enterica* (ANR_*Se*_) fall in Clade-2. ANRs from *Vibrio* (ANR_*Vibrio*_) are grouped in Clade-3 (Fig. 1A).

All predicted ANR members have a low molecular mass (4.36–9.54 kDa), and they exhibit 25–100% identity with Aar (Fig. 1B). In silico analysis of the MW of proteins in Clade-1 (sequences 1 to 14, Fig. 1B) and Clade-2 (sequences 19 to 46, Fig. 1B) shows that Clade-1 ANRs are smaller than Clade-2 ANRs, ranging from 7.233 to 7.787 kDa; mean 7.569 kDa and from 8.5 to 9.047 kDa; mean 8.772 kDa, respectively. Overall, members of Clade-1 also display the most significant amino acidic discrepancy between the three Clades. ANRs from Clade-2 are highly conserved among AE pathogens (100% coverage, 93.5% identity) (Fig. 1B). However, Rnr and ANR_*Vibrio*_ are the most distantly related ANRs to the archetype Aar, sharing only 25% identity.

### Characterization of the Rnr regulon in EPEC

**Figure 2:**
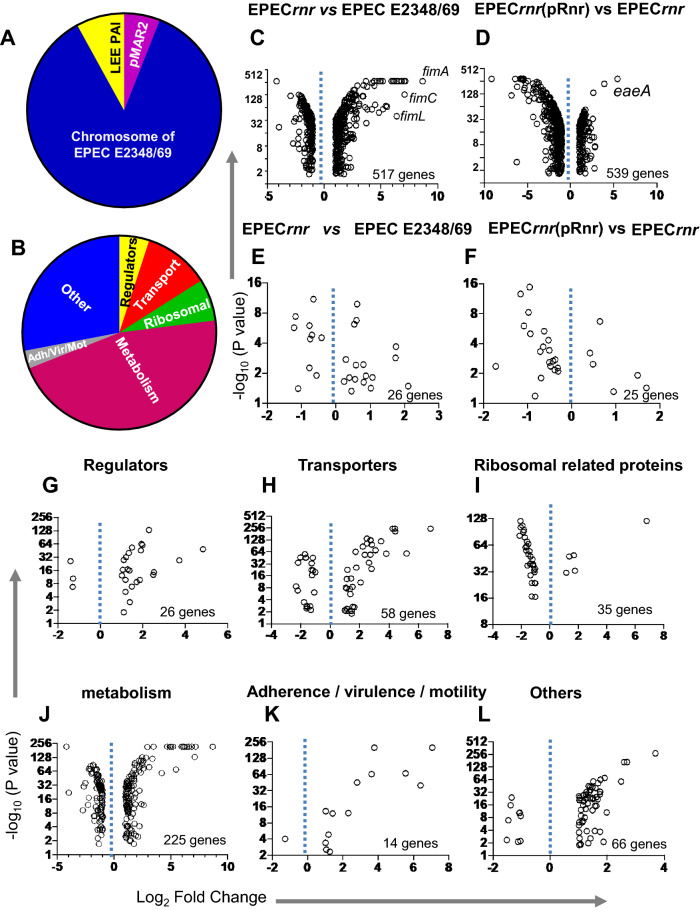
RNAseq analysis of the Rnr regulon. Comparison of differentially expressed genes for EPEC*rnr* vs. EPEC E2348/69 (**A**–**C**,**E**,**G**–**L**) and EPEC*rnr* vs EPEC*rnr* (pRnr) (**D**,**F**) are shown in volcano graphs based on P-value and fold change of differentially expressed genes (DEGs). DEGs are grouped into six main functional categories: regulators, transporters, ribosomal activities; metabolism; adherence/virulence/motility, and others (**B**,**G**–**L**). DEGs in the pMAR2 plasmid are shown in (**E**) and (**F**).

Since enteric pathogens use distinct mechanisms to colonize and invade their hosts, and the fact that the amino acidic identity between ANR members differs significantly among pathogens (Fig. 1B), it is uncertain whether ANR accomplishes the same regulatory function in different enteric pathogens. We sought to determine this gap in knowledge by dissecting the regulon and biological role of Rnr in attaching and effacing pathogens, which shares only ~ 25% of amino acid identity with the archetype Aar. Accordingly, the *rnr* gene was deleted in the prototype EPEC O127:H6 strain E2348/69, and RNA-seq determined its transcriptome. For these experiments, the wild-type (WT) EPEC, its isogenic EPEC*rnr* mutant, and EPEC*rnr* complemented with *rnr in-trans* [EPEC*rnr*(pRnr)] were grown in DMEM-high glucose for six hours to activate the expression of Rnr. Subsequently, total RNA from all strains was extracted and processed for cDNA synthesis, library construction, and DNA sequencing by CD genomics (NY, USA), as indicated in the material and methods. Bioinformatics analysis revealed approximately 500 genes that were differentially expressed (DEGs) in the Rnr regulon (± 1.5 fold, P < 0.05) (Fig. 2C–F). The majority of Rnr-regulated genes were located in the chromosome of EPEC E2348/69 (Fig. 2A) and associated with six major functional categories: genes involved in metabolism (46%), protein transport (11%), regulation (5%), ribosomal activities (7%), virulence (including bacterial adherence and motility) (3%), and other functions (28%) (Fig. 2B,G–L and Supplementary Fig. S1). 8% of Rnr-regulated genes were encoded in the LEE pathogenicity island located in the chromosome and 6% in the pMAR2 plasmid (GenBank FM180569.1) (Fig. 2A).

Ler and HNS were identified in the Rnr-regulon with 26 other regulatory proteins (Fig. 2G and Supplementary Fig. S1). Most of these regulators belong to the AraC/XylS family, including PerA, EutR, MelR, AdiY, YdiP, and GadX. Interestingly, we previously found that Aar also regulates GadX in response to the acid environment in EAEC^30,31^. Other important regulators under Rnr control are BssG, FimG, and CsgD, associated with biofilms or bacterial adherence (Fig. 2G).

In addition, we observed that a large number of genes regulated by Rnr are involved in metabolism (~ 200 genes), including the Rut operon (Supplementary Fig. S1), intricate in the degradation of exogenous pyrimidines as the sole nitrogen source, and the arginine succinyltransferase pathway which uses arginine as a source of carbon and nitrogen. Numerous genes of these operons are also under the NtrC control^32,33^.

### Rnr negatively regulates the locus of enterocyte effacement pathogenicity island (LEE-PAI)

**Figure 3:**
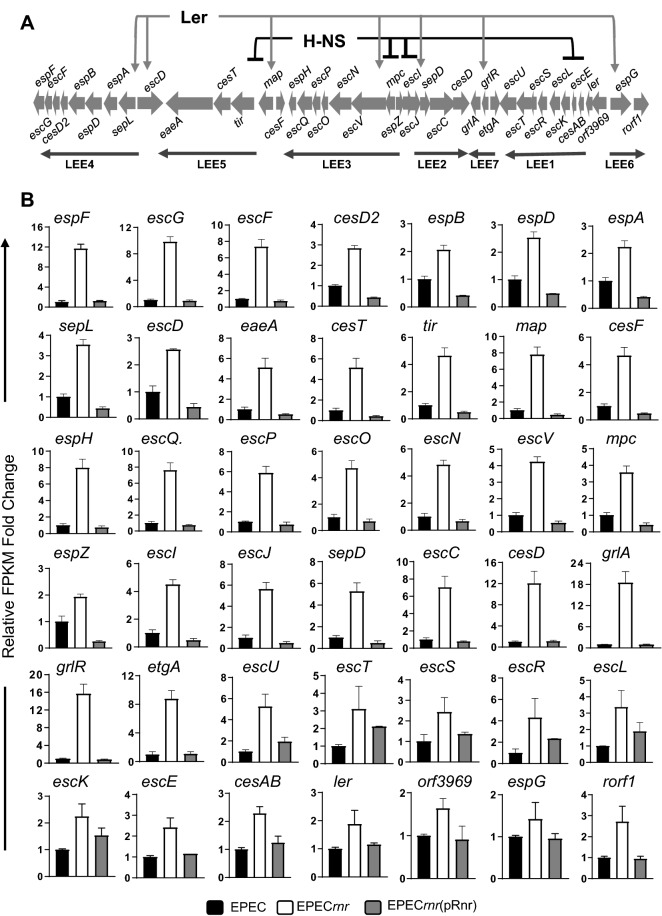
Rnr negatively regulates the locus of enterocyte effacement pathogenicity island (LEE-PAI). The LEE-PAI (~ 36 kb) is composed of 42 genes and seven operons encoding the T3SS (**A**). Transcriptional levels of Rnr-regulated genes were quantitated by RNA-seq in EPEC E2348/69 (black bars), EPEC*rnr* (open bars) and EPEC*rnr* (pRnr) (gray bars). The graph values represent relative FPKM fold changes with respect to the FPKM values of the WT strain.

The LEE-PAI (~ 36 kb) is composed of 42 genes and seven operons encoding the T3SS (Fig. 3A). Our transcriptomic data shows increased expression of *LEE* genes in EPEC*rnr*, whose complementation *in trans* with pRnr plasmid restored the expression of genes to comparable wild-type levels (Fig. 3B). Among those, twenty genes encode for proteins that form the core of the T3SS were at least ~ 2-fold upregulated, including proteins of the basal body (EscC, EscD, EscJ); inner membrane machinery (EscV, EscR, EscS, EscT, EscU); needle tip and translocon (EspD, EspB, EspA) and the EscN ATPse. The intimin gene (*eaeA*) and its receptor *tir* were also upregulated (~ 5-fold), which are associated with EPEC intimate adherence to host cells (Fig. 3B). Several LEE regulators (Ler, GrlA and GrlR) located in the LEE-PAI were upregulated (~ 2 to 18 fold) in EPEC*rnr* as compared to WT and complemented EPEC*rnr* strain (Fig. 3B).

**Figure 4:**
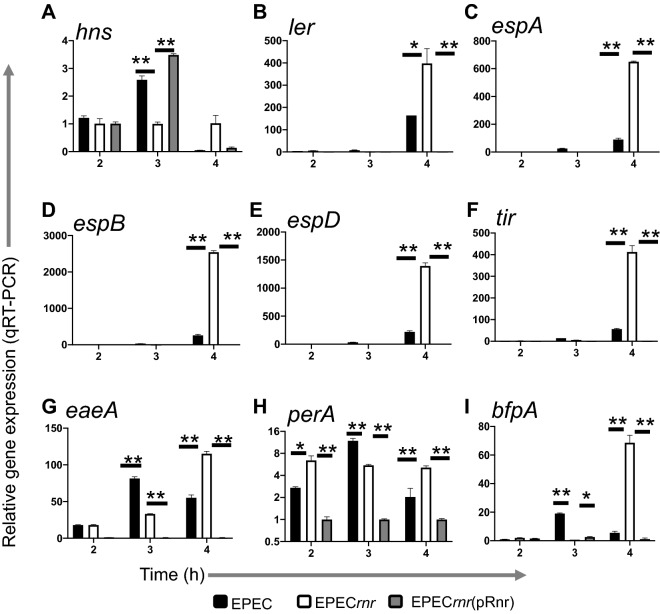
Validation of Rnr-regulated genes by qRT-PCR. EPEC E2348/69 (black bars), EPEC*rnr* (open bars), and EPEC*rnr* (pRnr) (gray bars) were inoculated in DMEM. Bacterial samples were obtained 2, 3, and 4 h post-inoculation for RNA isolation. Transcriptional levels of *hns* (**A**), *ler* (**B**), *espA* (**C**), *espB* (**D**), *espD* (**E**), *tir* (**F**), *eaeA* (**G**), *perA* (**H**), and *bfpA* (**I**) were quantitated by qRT-PCR. Expression levels for each queried gene were normalized to the constitutively expressed *rrsB* gene in EPEC. Data are representative of at least three independent experiments. Asterisks indicate significant differences by ANOVA (*P < 0.01; **P < 0.001).

To validate our transcriptome dataset, nine genes were selected based on their relevance in the virulence of EPEC for qRT-PCR analysis. We analyzed genes encoding structural proteins of the T3SS apparatus (*espA*,* espB*, and *espD*), regulatory proteins (*hns*, *ler*, and *perA*), and proteins involved in adherence (*tir*,* eaeA*, and *bfpA*). For qRT-PCR experiments, WT EPEC, EPEC*rnr*, and EPEC*rnr*(pRnr) strains were grown in DMEM-high glucose for 2, 3, and 4 h, and total RNA was isolated and prepared for gene expression analysis. Our data showed that 8 out of 9 analyzed genes exhibited higher levels of expression in the EPEC*rnr* mutant (~ 3-fold) compared to WT after 4 h of growth (middle log phase) (Fig. 4).

We previously found that Aar increases the expression of AAF genes in early EAEC growth stages by decreasing the expression of HNS, which acts as a repressor of AAF expression; however, when Aar is increased, it acts as a negative regulator of AAF by inactivating AggR, the positive AraC/XyS regulator of AAF^34^. Similar findings were observed in EPEC with *bfpA* and *eaeA* (Fig. 4G,I), which are regulated by PerA and HNS, respectively, and these, in turn, are regulated by Rnr (Fig. 4A,H).

**Figure 5:**
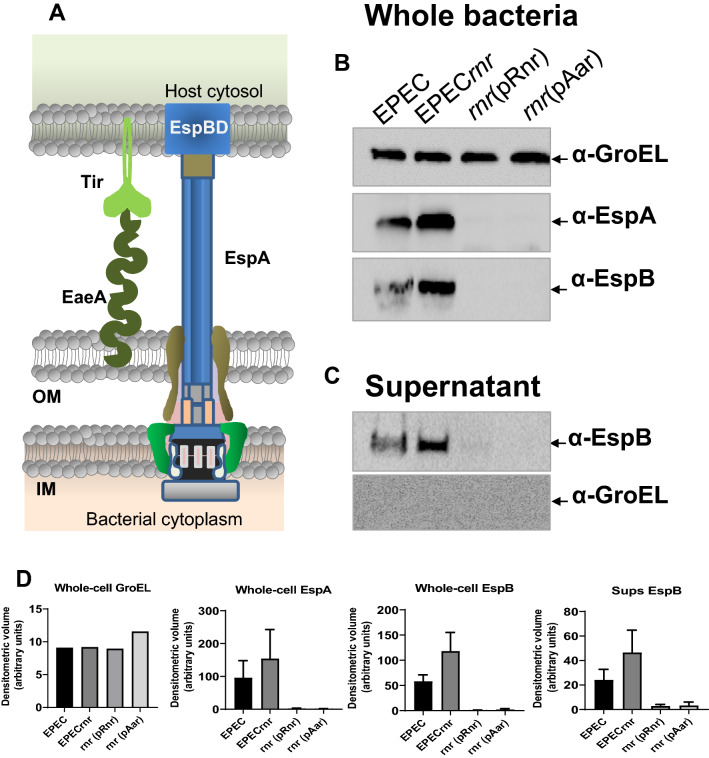
ANR controls the expression of EspA and EspB. The abundance of T3SS structural proteins EspA and EspB (**A**) were evaluated in whole-cell (**B**) and supernatant proteins (**C**) by Western blot. Densitometric quantitation of protein bands in Western blots from three independent images by ImageLab (Biorad) is displayed in (**D**). GroEL was used as an internal loading control for whole bacterial preps and as an indicator of cytoplasmic protein contamination in the secreted proteins fraction (**B**,**C**). Uncropped digital images are deposited in Supplementary Fig. S2.

We sought to determine if changes observed at the transcriptional level also correlated with changes at the protein level. Accordingly, EPEC derivatives were grown in DMEM until the late exponential growth phase (OD_600_ ηm of 1.0), and the abundance of T3SS structural proteins (EspA and EspB) (Fig. 5A) were evaluated in whole-cell (Fig. 5B) and supernatant proteins (Fig. 5C) by western blot using specific polyclonal antibodies for EspA, and EspB. GroEL was used as an internal loading control for whole bacterial preps and as an indicator of cytoplasmic protein contamination in the secreted protein fraction. In agreement with our transcriptomic data, we found that deletion of *rnr* correlated with an increased amount of EspA and EspB in whole bacterial and supernatant preps (Fig. 5B–D). In contrast, complementation of EPEC*rnr* with either pAar or pRnr plasmids drastically reduced the production of EspA and EspB in whole-cell preps and supernatants (Fig. 5B–D). Taken together, our findings indicate that members of the ANR family (Rnr and Aar) can regulate a variety of AraC/XylS and HNS regulators, including those controlling the LEE PAI in AE pathogens.

### Rnr protein directly interacts with HNS and Ler proteins

**Figure 6:**
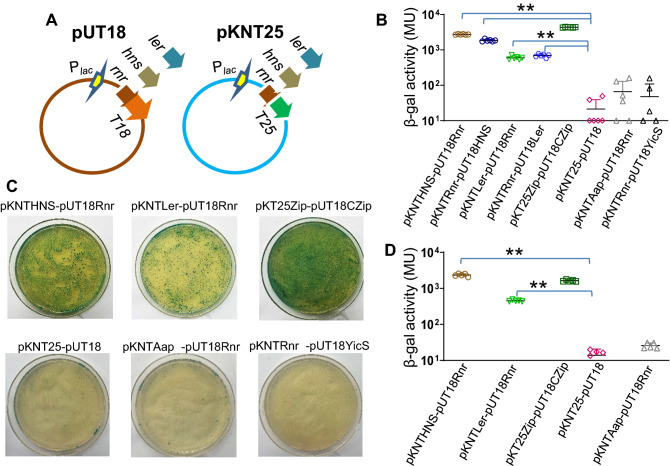
Rnr protein directly interacts with HNS and Ler proteins in the BACTH bacterial two-hybrid. pUT18 and pKNT25 fusion plasmids for Ler, HNS, and Rnr were generated (**A**) and cotransformed in *E. coli* BTH101 strain. Positive Rnr–HNS and Rnr–Ler interaction were confirmed on plates (**C**). The β-galactosidase activity was determined in the BACTH system for strains cotransformed with HNS/Rnr and Ler/Rnr derivatives. (**B**,**D**) Represent two independent experiments run with different BACTH clones. As controls, the *E. coli* BTH101 strain was co-transformed with either empty vectors (pKNT25 and pUT18) (negative control) or vectors encoding two irrelevant proteins (pKNTAap, pUT18YicS). Plasmids encoding the zip fragment (pKT25-zip and pUT18-zip) were used as a positive control in the BACTH system (**B**–**D**). Asterisks indicate significant differences by ANOVA (**P < 0.001).

We previously showed that Aar interacts with HNS global repressor affecting its regulatory activity^30^. Therefore, we sought to determine if Rnr can interact with HNS and Ler, both members of the HNS family. The Bacterial-two hybrid (BACTH) system is broadly used to scrutinize protein interactions between regulatory proteins^35^, and we have successfully used this approach to examine interactions between Aar-AggR and Aar-HNS^30,31^. Thus, we used the BACTH system to investigate interactions between Rnr, HNS, and Ler. Accordingly, *rnr*, *hns*, and *ler* genes were fused to T25 and T18 fragments of the catalytic domain of *Bordetella pertussis* adenylate cyclase, expressed in plasmids pKNT25 and pUT18, respectively (Fig. 6A)^35^. The resulting plasmids were co-transformed in different combinations of pUT18 and pKNT25 derivatives into the reporter strain *E. coli* BTH101. Remarkably, we observed protein–protein interactions of Rnr with members of HNS family; HNS, and Ler in the BACTH system manifested by the appearance of a moderate to intense blue color on agar plates (Fig. 6C) and quantification of the β-galactosidase activity (Fig. 6B,D). Taken together, our findings suggest that Rnr is regulating gene expression of the LEE-PAI by direct interaction with Ler and HNS global regulators.

### Rnr and Aar are interchangeable for regulating the T3SS in EPEC and fimbriae in EAEC

**Figure 7:**
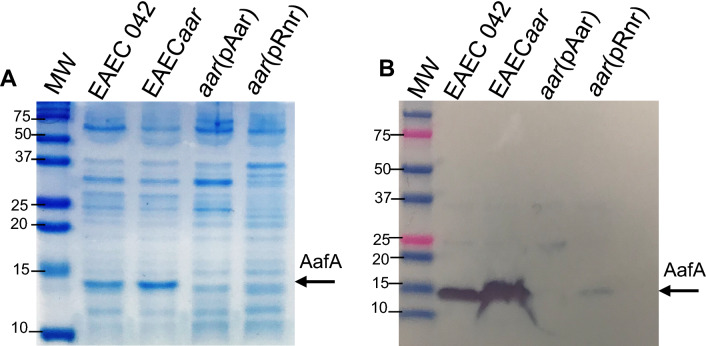
Rnr and Aar are interchangeable for regulating the fimbriae in EAEC. EAEC 042 derivatives were grown in DMEM-high glucose, and crude extracts of AafA were isolated and evaluated by SDS-PAGE (**A**) and Western blot (**B**) using anti-AafA antibody. Both pRnr and pAar plasmids showed similar inhibitory activity of AafA. Uncropped digital images of SDS-PAGE and Western blot experiments are deposited in Supplementary Fig. S2.

Since the heterologous expression of Aar in EPEC is capable of downregulating the expression of T3SS in EPEC in our previous experiment (Fig. 5B,C), we sought to determine whether the heterologous expression of Rnr in EAEC042 downregulates the expression of the AggR-regulated AAF, the main virulence factor of EAEC associated with host-interactions. Accordingly, we analyzed whole-cell proteins from EAEC derivatives expressing Aar and Rnr by SDS-PAGE and Western blot (Fig. 7). As expected, we found that Rnr was able to downregulate the expression of the major AAF fimbria subunit, AafA, in EAEC (Fig. 7), suggesting that despite the low homology between Aar and Rnr, they may possess structural features that allow function conservation between distantly related ANR members.

### Aar and Rnr negatively impact intestinal colonization in their respective pathogens

Despite the extensive molecular characterization of ANR in EAEC, its role in bacterial pathogenesis is not entirely understood, partly due to the lack of adequate animal models for *E. coli* pathogens. Since Aar downregulates the expression of AggR-regulated AAF in EAEC (Fig. 7)^29^, and Rnr downregulates PerA-regulated BfpA and genes associated with intimate adherence mediated by T3SS in EPEC (Fig. 4), we sought to determine the impact of ANR regulation in bacterial adherence and intestinal colonization.

**Figure 8:**
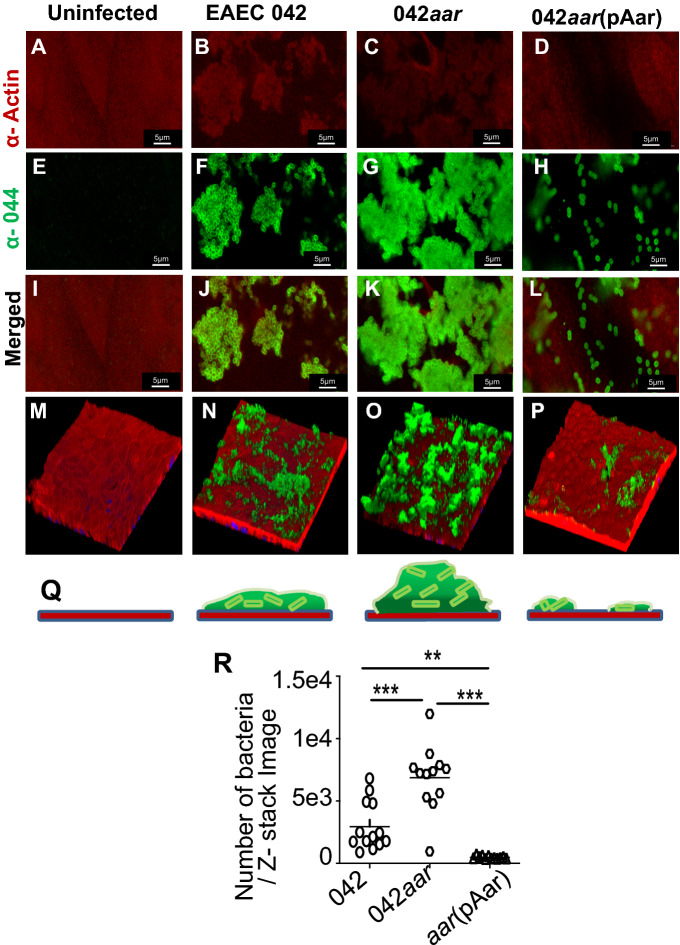
Aar regulates AAF and modulates colonization of the human intestine by EAEC. Human colonoids were infected with 042 (**B**,**F**,**J**,**N**); 042*aar* (**C**,**G**,**K**,**O**) or 042*aar* (pAar) (**D**,**H**,**L**,**P**). Uninfected cells serve as a negative control in the experiment (**A**,**E**,**I**,**M**). Anti-actin (for the cell) (**A**–**D**) and anti-O44 (for the bacteria) (**E**–**H**) antibodies were used in this study. The relative number of bacteria was measured in randomly selected Z-stack microscopic fields by ImageJ software (**R**). **Q** Illustrates the confocal microscopy results.

Human intestinal organoids have become the gold standard for studying host–pathogen interactions and have been successfully used to investigate essential features of EAEC and EPEC pathogenesis^36,37^. We, therefore, used this relevant intestinal model to examine the role of ANR in bacterial colonization. For these experiments, human intestinal colonoid monolayers were infected with parental 042, 042*aar*, and 042*aar* (pAar) at 37 °C for 6 h, and bacterial adherence was analyzed by confocal microscopy (Fig. 8A–P). We observed that the deletion of *aar* significantly increases bacterial colonization in human colonoids compared to the parental strain (Fig. 8C,G,K,O). In agreement with the negative role of Aar, complementation of 042*aar* with the pAar plasmid drastically reduced biofilm formation (Fig. 8D,H,L,P) as judged by the enumeration of bacterial cells on colonoids (Fig. 8R). Moreover, microscopic examination of bacterial biofilms revealed increased bacterial aggregation in colonoids infected with 042*aar* than parental 042 strain (Fig. 8N,O).

**Figure 9:**
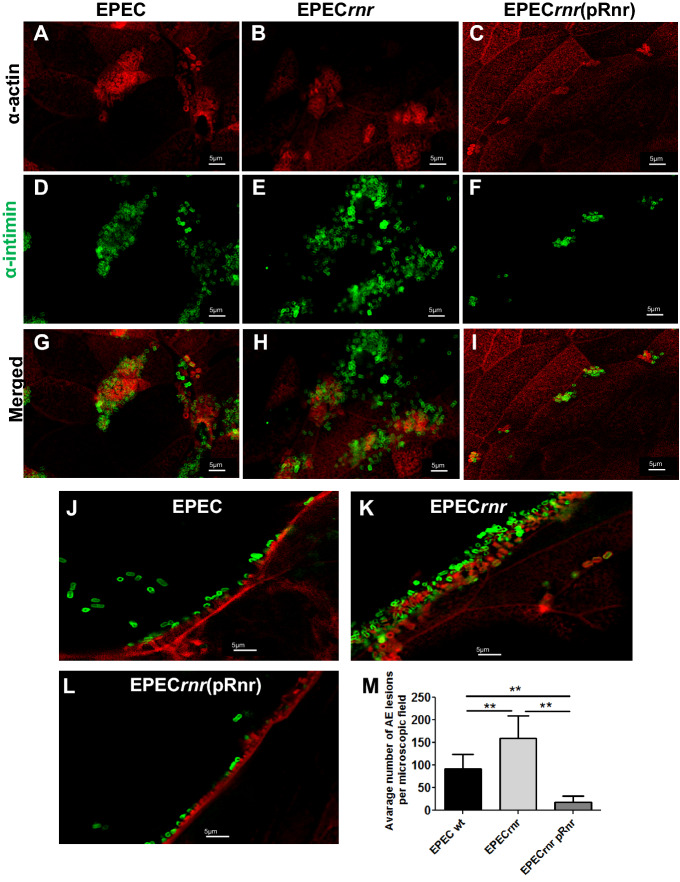
Rnr regulates AE lesion formation in human enteroids infected with EPEC. Human colonoids were infected with EPEC (**A**,**D**,**G**,**J**); EPEC*rnr* (**B**,**E**,**H**,**K**) or EPEC*rnr* (pRnr) (**C**,**F**,**I**,**L**). Anti-actin (for the cell) (**A**–**C**) and anti-intimin (for the bacteria) (**D**–**F**) antibodies were used in this study. Pedestal structures for EPEC (**J**), EPECrnr (**K**), and EPEC*rnr* (pRnr) (**L**). The relative number of bacteria was measured in randomly selected microscopic fields by ImageJ software (**M**).

We next determined whether Rnr impacts EPEC intestinal colonization and the formation of AE lesions in human intestinal organoids. Accordingly, human cell monolayers were infected with 10^6^ CFU of EPEC E2348/69, EPEC*rnr*, and EPEC*rnr*(pRnr) for 6 h at 37 °C. Subsequently, infected cells and uninfected controls were analyzed for EPEC adherence and formation of AE lesions by confocal microscopy (Fig. 9). The confocal images were pixel-quantified as previously reported (Fig. 9M)^36^. We observed a more significant number of adhering EPEC*rnr* strain on intestinal cell monolayers and which correlated with a greater number of AE lesions compared to the parental EPEC strain (Fig. 9A,B,D,E,G,H,M). Complementation of EPEC*rnr* with the pRnr plasmid drastically reduced bacterial adherence (Fig. 9C,F,I) and the number of AE lesions on intestinal cell monolayers as judged by actin polymerization beneath the adherent bacteria (Fig. 9L). Although AE lesions were observed in cells infected with all EPEC strains, cells infected with EPEC*rnr*(pRnr), which overexpresses Rnr, exhibit smaller actin pedestals than the WT or EPEC*rnr* strains as judged by qualitative analysis of confocal images (Fig. 9J,K,L). Taken together, our data suggest a central role of ANR in modulating intestinal colonization by diarrheagenic pathogens (Figs. 8, 9).

## Discussion

The ability of a pathogen to colonize a host and cause disease requires coordinated expression of genes that mediate nutrient acquisition, as well as genes involved in virulence. The mechanisms used by bacterial pathogens to regulate their fitness and virulence have been the subject of intense investigation for several decades. One such regulatory mechanism is exerted by the recently identified ANR family of small regulatory proteins whose mode of action is to interfere with the function of global regulators by protein–protein interactions, thereby modulating gene expression globally^27,29–31^. In EAEC, the prototype ANR termed Aar directly interacts with AggR and HNS proteins, resulting in an impaired ability of these regulators to bind DNA and, consequently, affecting the expression of hundreds of genes associated with metabolism, stress-response, fitness, and virulence^27,30,31^.

In this study, we sought to determine whether the regulatory properties of ANR are conserved in AE pathogens, such as EPEC, which has a multifaceted virulence scheme and whose ANR (Rnr) shares only 25% identity with Aar of EAEC. Accordingly, we defined the Rnr regulon of classical EPEC strain E2348/69 by comparing the transcriptomes of WT EPEC, isogenic EPEC*rnr*, and the complemented strain. We found that, like Aar in EAEC, Rnr regulates genome-scale gene expression in EPEC. More than 500 genes, including genes associated with fitness and virulence, were controlled by Rnr (Fig. 2). As expected, Rnr regulates the expression of HNS and multiple AraC/XylS regulators present in EPEC, including MelR, AdiY, YdiP, EutR, GadX, and PerA (Fig. 2G and Supplementary Fig. S1), which are associated with carbon metabolism, stress-response, and virulence functions, respectively (Fig. 2B). Even more remarkable is that Rnr negatively regulates the expression of the entire LEE pathogenicity island, including genes required for expressing the T3SS and its positive LEE-encoded regulator (Ler) (Fig. 3, Supplementary Fig. S1). Ler belongs to the HNS family, but unlike other HNS members, Ler activates the transcription of LEE genes by counteracting HNS-mediated repression^38^. We found that the expression of genes encoded in LEE2, LEE3, and LEE5 under the HNS-Ler control was downregulated by Rnr as judged by RNAseq and qRT-PCR transcriptional analysis (Figs. 3 and 4). Moreover, we observed that the abundance of T3SS-structural proteins EspA and EspB (encoded in LEE4) was moderately lower in whole-cell and supernatant preps of the WT strain when compared to EPEC*rnr* but drastically reduced in the complemented strain overexpressing Rnr (Fig. 5), highlighting the negative regulatory role of Rnr.

On the other hand, we confirmed the direct protein–protein interaction of Rnr with Ler and HNS in vivo using the BACTH system, which has been successfully used to test interactions between regulatory proteins, including Aar^27,30,31^. Since Rnr binds to both a silencer (HNS) and anti-silencer (Ler), and the fact that binding of Aar to HNS and AggR in EAEC hampers the function of these regulators, we have envisioned two possible mechanisms that may explain the negative regulatory role of Rnr on the expression of LEE genes: (1) direct Rnr interaction with Ler may inhibit its positive feedback activity on the LEE PAI and (2) since HNS function as a repressor of its transcription, binding of Rnr to HNS may inhibit the HNS silencing effect on its promoter, favoring HNS expression, and consequently, the repression of LEE genes. Release of the silencing effect of HNS on its promoter was previously demonstrated for Aar in EAEC^30^. We are currently characterizing the hierarchy of Rnr-interactions in the context of HNS and AraC/XylS regulators in space and time to ascertain the regulatory landscape of Rnr.

Interestingly, AraC/XylS and HNS are critical regulators of fimbriae and pili in most pathogens. These essential organelles allow bacteria to colonize their hosts and persist on abiotic surfaces. In EAEC, the AraC family member AggR controls the expression of AAF^23,24^. In ETEC, the expression of many types of adhesive pili depends on the AraC member Rns^39^. In contrast, ToxT, an AraC member from *V. cholera*, regulates the transcription of genes encoding the two major virulence factors, the toxin-coregulated pilus (TCP) and cholera toxin (CT)^40^. Unlike EAEC, EPEC relies on virulence factors that promote LA and intimate contact with the epithelium mediated by a T3SS, which results in attaching and effacing (AE) lesions, the hallmark of EPEC infection. The LA phenotype is determined by the BFP, which is regulated by PerA (a member of the AraC/XylS family)^41–43^. In this study, we show that Rnr controls the expression of PerA and the expression of the adhesion protein intimin and its receptor Tir (Fig. 4).

**Figure 10:**
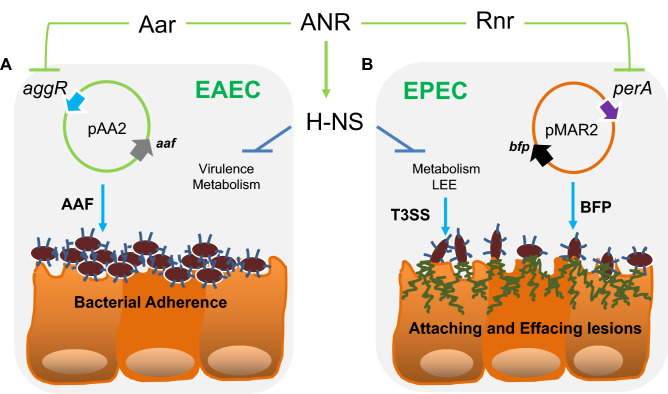
Hypothetical model of ANR regulation of intestinal colonization by enteropathogens.

Most importantly, we found that both Aar and Rnr impact EAEC and EPEC colonization of human intestinal organoids (Figs. 8 and 9). Although ANR members may impinge on multiple regulons within the same bacterial cell to modulate multiple virulence traits, ANR’s primary function could be perhaps the modulation of colonization dynamics of enteric pathogens in the intestine through repression and anti-repression of AraC/XylS and HNS during pathogen–host interactions, and that this process may enable bacteria to spread out and persist in the host. Additional studies in suitable animal models should be conducted to test this hypothesis. In summary, our findings suggest that despite the low homology between ANR cognates in pathogens with different pathogenic schemes, ANR is a highly conserved mechanism of regulation of AraC/XylS and HNS regulators in diarrheagenic bacteria (Fig. 10).

## Materials and methods

### Bacterial strain and growth conditions

Bacterial strains and plasmids used in this study are listed in Table 1. 042*aar* was previously generated in our laboratory^29^. The *rnr* locus (2,721,409–2,721,642, GenBank FM180568.1) in EPEC E2348C_2635 was replaced with kanamycin (km) by lambda red recombination^44^. Our strategy, replaced the *rnr* locus with the Km gene from start to stop codon to avoid polar effects in downstram genes. Briefly, EPEC(pKD46) was grown in 100 ml of LB at 30 °C to an OD_600_ ηm of 0.4. The lambda red enzyme was induced with 20 mM of l-arabinose for 1 h. The culture was centrifuged, and the bacterial pellet was prepared for electroporation using sterile water. 100 µl of electrocompetent cells were mixed with ~ 500 ng of DNA and electroporated using a Gene Pulser Xcell Bio-Rad electroporator. Cells were recovered in 3 ml of SOC medium at 37 °C for 3 h. The DNA used for the electroporation was amplified by PCR using EPEC as a template and specific *rnr* flanking primers (Table 2). Positive strains were identified by PCR and DNA sequencing. EAEC 042 and EPEC E2348/69 derivatives were routinely propagated in Luria Broth (LB) and Dulbecco’s modified Eagle’s medium (DMEM). For growth in DMEM, strains were first cultivated overnight in LB with appropriate antibiotics and diluted 1:100 (for EAEC) or 1:50 (for EPEC) in DMEM with 0.4% glucose (DMEM-HG) (Gibco, Grand Island, NY) as previously described^23^.

**Table 1:** Strains and plasmids used in this study.

Strain	Genotype or description	Source
EAEC strain 042 derivatives		
EAEC strain 042	WT EAEC, Cml^r^	Lab collection
EAEC 042*aar*	EAEC 042 mutant in *aar*, Cml^r^, Kan^r^	Santiago et al.^29^
EAEC 042*aar*(pAar)	EAEC*aar* mutant complemented *in trans* with pAar	Santiago et al.^29^
EAEC 042*aar*(pRnr)	EAEC*aar* mutant complemented *in trans* with pRnr	Santiago et al.^29^
EPEC strain E2348/69 derivatives		
EPEC strain E2348/69	WT EPEC, Str^r^	Lab collection
EPEC*rnr*	EPEC strain E2348/69 mutant in *rnr*, Str^r^, Kan^r^	This study
EPEC*rnr* (pRnr)	EPEC*rnr* mutant complemented *in trans* with pRnr	This study
EPEC*rnr* (pAar)	EPEC*rnr* mutant complemented *in trans* with pAar	This study
*E. coli* strain BTH101 derivatives		
BTH101	*E. coli* reporter strain for BACTH system	Euromedex
BTH101 pKT25/pUT18C	Negative *E. coli* reporter strain for BACTH system	This study
BTH101 pKT25Zip/pUT18CZip	Positive *E. coli* reporter strain for BACTH system	This study
BTH101 pKNT25-HNS/pUT18-Rnr	*E. coli* BTH101 co-transformed with pKNT25-HNS/pUT18-Rnr	This study
BTH101 pKNT25-Ler/pUT18-Rnr	*E. coli* BTH101 co-transformed with pKNT25-Ler/pUT18-Rnr	This study
BTH101 pKNT25-Rnr/pUT18-HNS	*E. coli* BTH101 co-transformed with pKNT25-Rnr/pUT18-HNS	This study
BTH101 pKNT25-Rnr/pUT18-Ler	*E. coli* BTH101 co-transformed with pKNT25-Rnr/pUT18-Ler	This study
BTH101 pKNT25-Aap/pUT18-Rnr	*E. coli* BTH101 co-transformed with pKNT25-Aap/pUT18-Rnr	This study
BTH101 pKNT25-Rnr/pUT18-YicS	*E. coli* BTH101 co-transformed with pKNT25-Rnr/pUT18-YicS	This study

**Table 2:** Primers used in this study (Primer (5 → 3).

Name	pRnr derivatives
Rnr NdeI sense	CGTCATCATATGCATCAGGATATCAAAGAGTACAGAGCCGGA
Rnr XbaI rev	CGTCATTCTAgATTAAAGTTCTTCGCATTCATGGCGTGGCCTCCA
qRT-PCR primers	
RrsB sense	TCCAGGTGTAGCGGTGAAAT
RrsB rev	TGAGTTTTAACCTTGCGGCC
Ler sense	CGAGAGCAGGAAGTTCAAAGT
Ler rev	GCCCTTCTTCATTGCGGTAG
EspA sense	AGGCATCTAAGGAGTCAACCA
EspA rev	CCAGCGCCTAATTGAGCATT
EspB sense	CTAAAGGCGCGAGTGATGTC
EspB rev	TCTGAGCCGAAGTAGCAACA
EspD sense	GAGATCTACGCGGATGGACA
EspD rev	CGCCCATAACATCAACTGCA
Tir sense	AAGTGCAGGCAGATGGTACT
Tir rev	ATTTTCGTACGAGCTTCCGC
eaeA sense	ATTCCTCTGGTGACGATGGG
eaeA rev	ATCGTAACGGCTGCCTGATA
BfpA sense	AGTAATGAGCGCAACGTCTG
BfpA rev	ACATGCCGCTTTATCCAACC
PerA sense	TGCGAACCTCAATGAAATGCA
PerA rev	ACCCTGTCTACGATGCTCTT
HNS sense	ATAGCCTTGCTGCCGTTAAA
HNS rev	CGAGGGATTTACCTTGCTCA
BACTH primers	
Ler HindIII sense	ATGCGATCCAAGCTTGATGCGGAGATTATTTATTATGAATATGGAAACTAATTCACATA
Ler BamHI rev	ATGCGATGGGATCCATATTTTTCAGCGGTATTATTTCTTCTTCAGTGTCCTTCACAAG
HNS HindIII sense	ATGCGATCCAAGCTTGATGAGCGAAGCACTTAAAATTCTGAACAACATCCGTAC
HNS BamHI rev	ATGCGATGGGATCCTGCTTGATCAGGAAATCGTCGAGGGATTTACCTTGC
Rnr HindIII sense	ATGCGATCCAAGCTTGATGCATCAGGATATCAAAGAGTACAGGGCTGGGAACCG
Rnr BamHI rev	ATGCGATGGGATCCAGCGTTTCGCGTTCATGAGGCGGCTTCCATCC
Lambda red primers for generation of EPEC*rnr* (FM180568.1)	
$\lambda$ Rnr sense	AATAACGCCATGTCGCTGATTGGTGAAGCCGTCCAGGTAATTGGCAGCAAAAGCTATATCCGCGTGTATGAGCGCGTCGGTGATTCTGCTGAATACCGCGCAATCCCGCTTGATATTGCAGGGGTTTAACATGCATCAGGATATCAAAGAGTACAGGGCTGGGAACCGTTGCGCAGCGTAGTGTAGGCTGGAGCTGCTTC
$\lambda$ Rnr rev	CTCGACATTAATTTCAACCACACAACCACATTTAAAATCTCTGGCAGGTGCAACAGTTTTTACCACCCGACCACCACGCACTGCCCGATGTGCAATGTGCATGAAGCGAGTCCCTGGCGGATATAGCTGATTAAAGCGTTTCGCGTTCATGAGGCGGCTTCCATCCTTTCAGGTGAGCATATGGGAATTAGCCATGGTCC

### RNA-seq

RNA was extracted from EPEC E2348/69 derivatives (WT EPEC, EPEC*rnr*, and EPEC*rnr*(pRnr) grown in DMEM-HG as previously reported^23,30^. RNA was extracted with TRIzol, DNA was removed, and Illumina stranded RNA-seq library was prepared by CD genomics (NY, USA). Isolated RNA samples were subjected to RNA sequencing (RNAseq) by utilizing Illumina NovaSeq 6000 platform to generate paired-end reads at CD genomics. Reads were mapped to the EPEC strain E2348/69 chromosome (FM180568.1) and pMAR2 plasmid (FM180569.1) with the BWA aligner^45^. Counts for each annotated genomic feature were determined by htseq-count (http://www-huber.embl.de/users/anders/HTSeq/doc/count.html). Differential expression between counts for each feature was then calculated with DESeq^46^ using the false-detection rate-adjusted Benjamini Hochberg P value. The P-value obtained from the test was corrected, and the false discovery rate (FDR) was used as a key indicator of differentially expressed genes. The fold change of differentially expressed genes vs. P value was plotted using GraphPad Prism 6 (GraphPad Software, Inc., CA, USA). During the analysis, fold change ≥ 2 and FDR < 0.05 were set as screening criteria. Fold change indicates the ratio of expression levels between the samples.

### Real-time quantitative reverse transcription-PCR (qRT-PCR)

Overnight bacterial cultures of EPEC E2348/69 derivatives were diluted 1:50 in 13 ml of DMEM-HG (ANR-inducing conditions) and incubated at 37 °C without shaking for 2, 3, and 4 h. Extraction of RNA, cDNA synthesis, and qRT-PCR assays were performed as previously described^23^. Primers for the qRT-PCR analysis are reported in Table 2. Reactions were run in experimental duplicate using two independent cDNA preparations. Expression levels for each queried gene were normalized to the constitutively expressed *rrsB* of EPEC as previously described^23,47^.

### Detection of AAF/II, EspA, and EspB

To detect the major fimbria subunit of AAF/II (AafA), strains were grown in 13 ml of DMEM-HG to reach an OD_600_ ηm of 0.8. Bacteria were pelleted, resuspended in 100 µl of 0.5 mM Tris, 75 mM NaCl and heated for 30 min at 65 °C. AafA was analyzed in heat-prep supernatants by SDS-PAGE and Western blot analysis. Protein samples were separated in acrylamide gels and transferred to Immobilon-P membranes (BioRad, Hercules, CA, USA) using standard protocols. Membranes were incubated overnight with a polyclonal anti-AafA antibody. The next day, membranes were washed twice in PBS-0.1% tween and incubated for 1 h with horseradish peroxidase-conjugated goat anti-rabbit IgG antibody (ThermoFisher). Membranes were washed thrice with PBS-0.1% tween and imaged using ChemiDoc Imaging System (BioRad).

Expression of EspA and EspB was analyzed in whole-cell and supernatants of EPEC cultures by Western blot. Briefly, EPEC derivatives were grown overnight in LB, diluted 1:50 in 13 ml of DMEM-HG, and grown at 37 °C to an OD_600_ ηm optical density of 1.0. The cultures were centrifuged at 20,000×*g* for 5 min, and bacterial pellets were prepared for Western blot analysis. On the other hand, supernatants were collected and filtered through a 0.22 μm filter (Millipore). The supernatants were precipitated with 10% trichloroacetic acid overnight at 4 °C and centrifuged at 18,000×*g* for 15 min. Protein pellets were washed with 1 ml of acetone and dissolved in SDS-PAGE sample buffer saturated in Tris. Proteins were analyzed by Western blot using polyclonal anti-EspA and anti-EspB antibodies (kindly donated by Dr. James Kaper and Jane M Michalski, University of Maryland). Cytoplasmic GroEL (Abcam) was detected as an internal loading control and as an indicator of cytoplasmic protein contamination in the secreted protein fraction.

### Bacterial adenylate cyclase two-hybrid system (BACTH)

The genes *ler*, *hns*, and *rnr* from EPEC were amplified by PCR and fused to the T25 (pKNT25 derivatives) or T18 (pUT18 derivatives) domain of *Bordetella pertussis* CyaA as previously reported^29,35^. Plasmids pKT25/pUT18C and pKT25Zip/pUT18CZip were used as experimental negative and positive controls, respectively. We also expressed two irrelevant proteins (Aap and YicS) in the BACTH system as additional negative controls for the interacting experiments with Rnr. The primers used in this work are listed in Table 2. The plasmids were purified and cotransformed into the reporter strain *E. coli* BTH101. Colonies were selected on LB agar plates containing carbenicillin (100 µg/ml), kanamycin (50 µg/ml), 5-bromo-4-chloro-3indolyl-β-d-galactopyranoside (X-Gal) (40 µg/ml), and isopropyl-β-d-thiogalactopyranoside (IPTG) (1 mM).

The clones were grown at room temperature for 48–72 h in LB plates with 1 mM IPTG. β-Galactosidase assays were performed accordingly to the method of Miller^48^. Briefly, bacterial samples were suspended in 1 ml of Z buffer (60 mM Na_2_HPO_4_·7H2O, 40 mM NaH_2_PO_4_·H2O, 10 mM KCl, 1 mM MgS0_4_·7H_2_O, 50 mM β-mercaptoethanol), 20 µl of 0.1% SDS and 40 µl of chloroform. 100 µl of the sample was incubated with 20 µl of ONPG (4 mg/ml) for 2 min at room temperature. The reaction was terminated by the addition of 50 µl of 1 M Na_2_CO_3_. Samples were diluted in 800 µl of Z-buffer. Optical densities at 420, 550, and 600 were determined. β-galactosidase activity was calculated by using the Miller formula (Miller unit = 1000 × (Abs_420_ − (1.75 × Abs_550_)/T × V × Abs_600_); T, reaction time; V, volume of culture assayed in milliliter).

### Human intestinal organoid culture

Human intestinal organoids used in this study were derived from a colonoid/enteroid repository previously established from deidentified biopsy specimens from healthy subjects who provided written informed consent at Johns Hopkins University by approved guidelines and regulations (IRB NA_00038329).

The maintenance of human organoids and preparation of colonoid monolayers were previously described^36^. Briefly, organoids were routinely cultured as 3D cysts embedded in Matrigel (Corning) and used to prepare cell monolayers in 24-well, 0.4 µm pore size polyester membrane cell culture inserts (Transwell supports, Corning) precoated with 100 µl of 34 µg/ml of human collagen IV solution (Sigma). Intestinal monolayers were routinely grown at 37 °C with 5% CO_2_ until confluency as assessed by the increase in transepithelial electrical resistance (TEER), measured using an epithelial volt/ohm meter (EVOM, World Precision Instruments). Confluent monolayers were differentiated for five days before infections.

### Confocal microscopy

EAEC 042 and EPEC E2348/69 derivatives were grown overnight in LB supplemented with appropriate antibiotics (Sigma Chemical Co, St. Louis, MO). The next day, overnight cultures were diluted 1:50 (V/V) in DMEM-HG medium (Invitrogen, USA) and incubated at 37 °C with shaking to the mid-log phase (OD_600_ = 0.6) to induce ANR expression. Bacterial cultures were adjusted to 10^8^ CFU/ml in PBS, and 10 µl (10^6^ CFU) was added to the apical surface of colonoid monolayers. Cells were infected for six hours at 37 °C, 5% of CO_2_. Following the bacterial infection, the cells were fixed with Carnoy’s solution (90% methanol, 10% glacial acetic acid), washed three times with PBS, permeabilized with 0.1% saponin, and blocked with 2% bovine serum albumin/fetal bovine serum for 30 min (Sigma Aldrich, USA). Cells were rinsed with PBS and incubated overnight at 4 °C with primary antibodies diluted 1:100 in PBS containing 15% FBS and 2% BSA. As primary antibodies, we used anti-O44 for EAEC staining, anti-intimin for EPEC staining, and Alexa-phalloidin for cell-actin staining. Stained cells were washed three times with PBS, followed by incubation with appropriate Alexa-conjugated secondary antibodies (Molecular Probes/Invitrogen, USA) diluted 1:500 in PBS. Hoechst (Vector Laboratories, USA) was used at a 1:1000 dilution in PBS for nucleus/DNA labeling. After incubation, cells were washed three times for 5 min each and mounted in ProLong Gold (Vector Laboratories, USA) overnight at 4 °C. Confocal imaging was carried out at the Imaging Core Facility at the University of Virginia using an LSM-710 Multiphoton laser-scanning confocal microscope (Zeiss, Germany) running ZEN 2012 (black and blue edition) imaging software (Zeiss, Germany). Images were captured with a 64 × oil objective. The same settings were used to image across samples for quantitative analysis of bacteria and AE lesions (actin polymerization). At least 10 Z-stack images of 2-μm intervals up to a depth of 20 μm for each treatment were obtained from three independent experiments. The Z-stack image depth corresponded to a detectable green fluorescent signal (bacteria) on colonoids stained for actin. A robust fluorescent signal (polymerized actin) corresponds to AE lesion formation in EPEC-infected bacteria. The relative number of bacteria (or AE lesions) was measured in randomly selected Z-stack microscopic fields by ImageJ software (NIH), using the particle enumeration algorithm as previously reported^36^. Briefly, single images from Z-stacks exhibiting green fluorescent bacteria or fluorescent polymerized actin (AE lesions) were obtained using Blue edition ZEN2012 software Zeiss, (Zeiss, Germany). Images were opened as 16-bit type images with ImageJ. Threshold values were adjusted to eliminate the background. Bacteria or AE lesions (particles) were enumerated in images processed as Binary > Watershed images. This algorithm separates particles that are close together (e.g., aggregated bacteria). Lastly, images were analyzed as particles set as size (pixel2) = I0-infinite, which is relatively close to the size of *E. coli* in 64 × confocal images. Particle (bacteria or AE lesions) counts in each image of a Z-stack were input in an Excel sheet and plotted using the Prism software (Graph Pad).

### Bioinformatic and statistical analysis

The amino acid homology of ANR proteins was obtained using Clustalw algorithms (http://www.genome.jp/tools/clustalw/). Values are presented as mean ± and standard error of the mean (SEM). Statistical significance was determined using analysis of variance (ANOVA) with Bonferroni’s post-test (Prism GraphPad) to compare groups, including a minimum of n = 3 replicates. Results were considered significant at P < 0.05.

### Institutional guidelines

Human intestinal organoids used in this study were derived from a colonoid/enteroid repository previously established from deidentified biopsy specimens from healthy subjects, who provided written informed consent at Johns Hopkins University (Protocol NA_00038329). All methods were carried out in accordance with the University of Virginia-approved guidelines and regulations (IRB-HSR # 18959). All experimental protocols were approved by the University of Virginia Review Board (IBC number: 1329-11).

## Supplementary Information

Supplementary Figure S1.

Supplementary Figure S2.

## Data Availability

The RNA-seq data discussed in this publication have been deposited in NCBI’s Gene Expression Omnibus and are accessible through GEO accession number: GSE225626. To review GEO accession GSE225626: go to https://www.ncbi.nlm.nih.gov/geo/query/acc.cgi?acc=GSE225626. Enter token yhczigeijvsnnir into the box.
