# Supplementary material for: Highly-conserved regulatory activity of the ANR family in the virulence of diarrheagenic bacteria through interaction with master and global regulators
Source: Sci Rep. 2023 Apr 29;13:7024. doi: 10.1038/s41598-023-33997-0 (PMC10148876; doi:10.1038/s41598-023-33997-0)
Supplement: Supplementary file 2 — Supplementary Figure S2. [file 41598_2023_33997_MOESM2_ESM.pdf]

Highly-conserved regulatory activity of the ANR family in the virulence of diarrheagenic bacteria through interaction with Master and Global regulators

Diana Rodriguez-Valverde, Jorge A. Giron Yang Hu, James P. Nataro, Fernando Ruiz-Perez and Araceli E. Santiago

GroEL in cell lysates

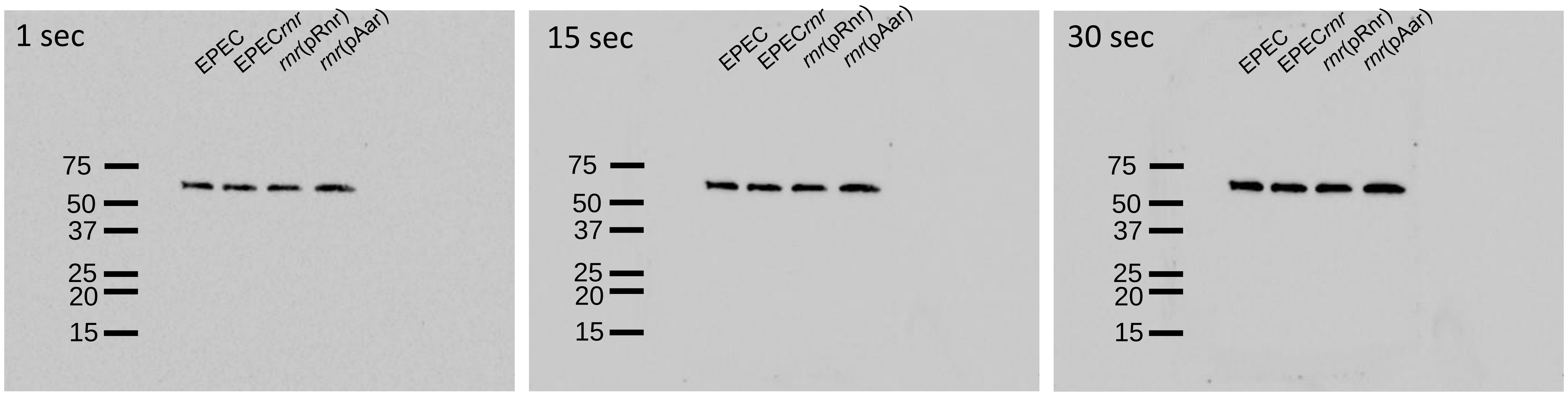

EspA in cell lysates

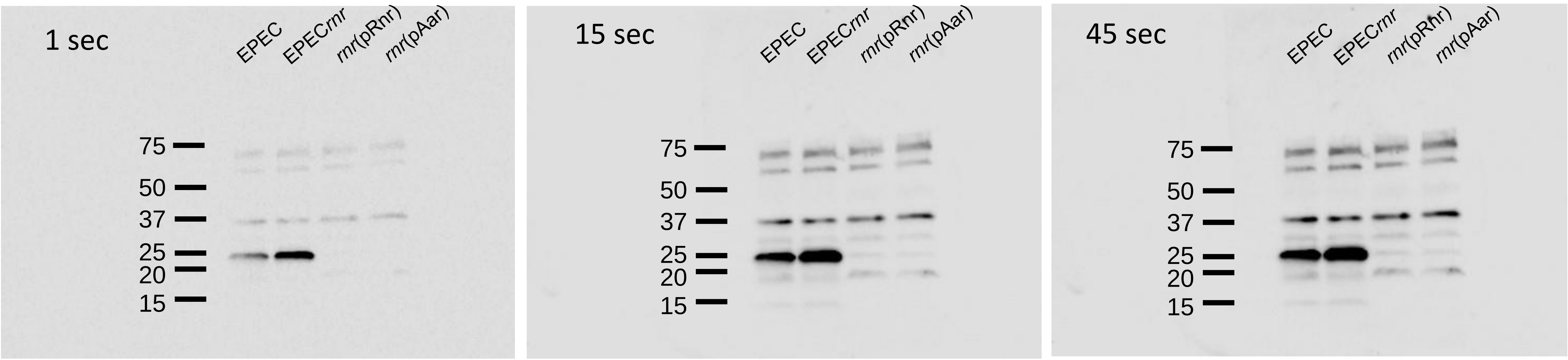

EspB in cell lysates

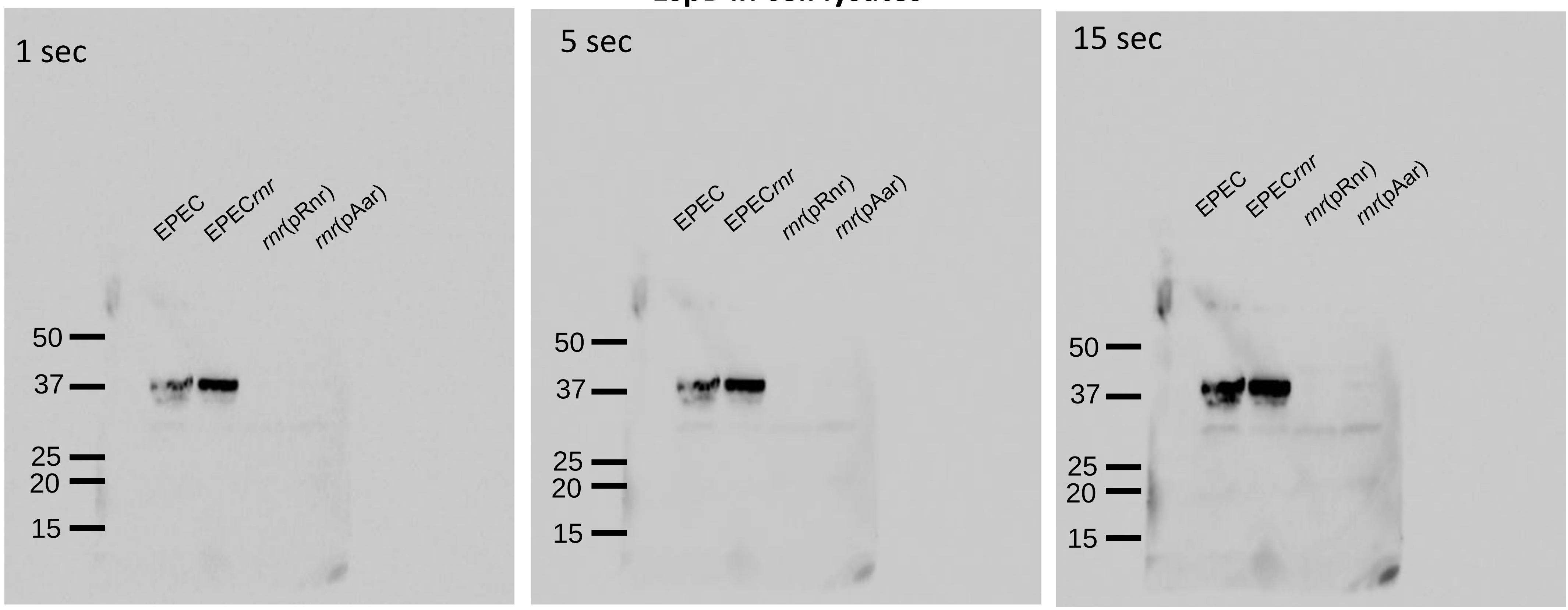

EspB in supernatants

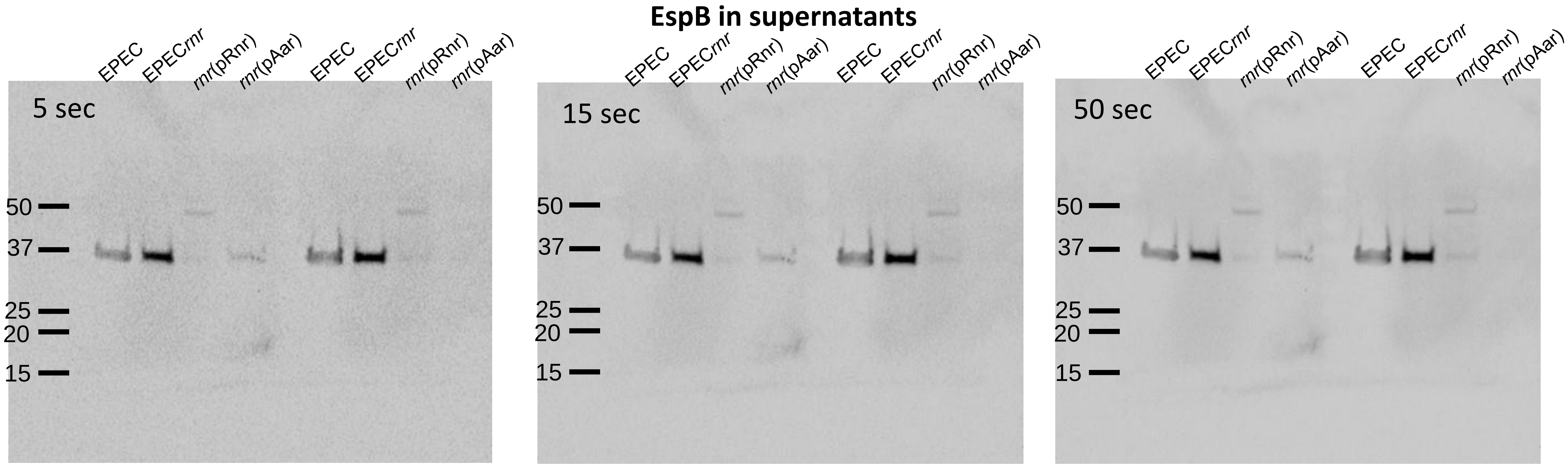

Uncropped digital images of Western blot experiments depicting EspA, EspB, and GroEL in Figure 5 of the main manuscript. Membranes were probed with polyclonal antibodies against EspA, EspB, or GroEL. Membranes were exposed at the specified times for chromogenic detection. Samples for EspB in supernatants (bottom) were run in duplicates on the same gel/membrane.

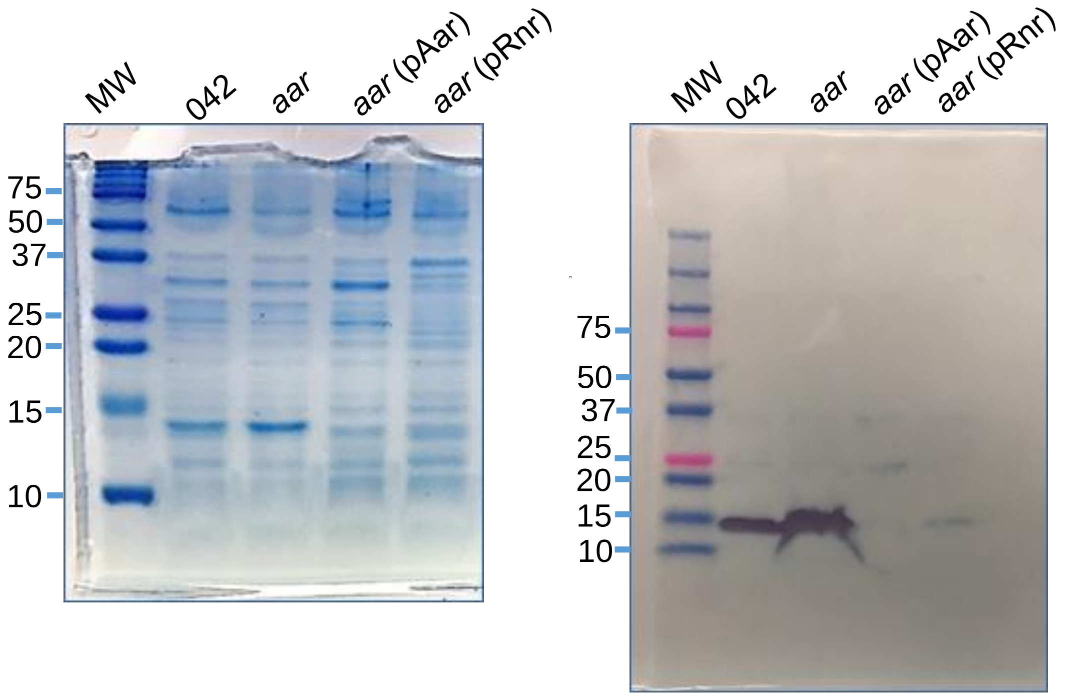

Uncropped digital images of SDS-PAGE and Western blot experiments depicting AafA in Figure 7 of the main manuscript.
